# Can Stem Cells Improve Left Ventricular Ejection Fraction in Heart Failure? A Literature Review of Skeletal Myoblasts and Bone Marrow-Derived Cells

**DOI:** 10.7759/cureus.11598

**Published:** 2020-11-20

**Authors:** Meghan M Cheung, Nusrat Jahan

**Affiliations:** 1 Family Medicine, California Institute of Behavioral Neurosciences & Psychology, Fairfield, USA; 2 Internal Medicine, California Institute of Behavioral Neurosciences & Psychology, Fairfield, USA

**Keywords:** heart failure, heart failure therapy, left ventricular ejection fraction, stem cell therapy, skeletal myoblasts, bone marrow derived stem cells, stem cells transplantation, bone marrow, cd34+, stem cells

## Abstract

Heart failure is a life-threatening condition that affects millions worldwide and is only expected to get worse with an ageing population. Current treatment regimens rely on medical therapy and heart transplantation as a last resort. Stem cells have been undergoing clinical trials worldwide as a hope for a new and safe clinical treatment. Skeletal myoblasts and bone marrow-derived stem cells are two types of stem cells being tested. The objective is to evaluate the efficacy of these two types of stem cells for heart failure therapy. Data were searched in PubMed using both regular and Medical Subject Heading (MeSH) keywords (stem cells, therapy, heart failure) and then filtered using inclusion/exclusion criteria (language, species, publication date, and age). In total, 31 research articles were reviewed (14 clinical trials, four randomized control trials, nine review articles, one case report, one comparative study, one systematic review, and one categorized as a systematic review and meta-analysis). Both skeletal myoblasts and bone marrow-derived stem cells showed mixed results in improving left ventricular ejection fraction in heart failure patients in the majority of studies. Larger studies need to be done to further investigate the efficacy of stem cells as a therapy for heart failure.

## Introduction and background

With approximately 26 million people diagnosed worldwide, heart failure has become an ever-increasing strain and burden on the healthcare system and on society. With an ageing population and no cure, this number is only expected to increase [1]. Heart failure is a life-threatening clinical condition where damage to the heart causes problems in ventricular filling or in the ejection of blood. Common clinical symptoms include dyspnea, fatigue, and edema. Left ventricular ejection fraction (LVEF) can show the functional status of the heart and be used to clinically classify heart failure. A reduced LVEF and an LVEF <45% are predictors of poor outcome and increased mortality in an inpatient setting, respectively [2]. A common classification used for heart failure is the New York Heart Association functional classification: NYHA I (no limitations to physical activity), NYHA II (mild symptoms to ordinary physical activity), NYHA III (comfortable at rest, marked symptoms with less than ordinary activity), and NYHA IV (severe limitations, symptoms at rest) [2]. 

Management of heart failure is primarily in the form of drug therapy with the aim of improving prognosis and symptoms and reducing mortality and morbidity. Medical management depends on patient history along with signs and symptoms of heart failure and includes diuretics, angiotensin-converting enzyme inhibitors or angiotensin receptor blockers, beta-adrenergic blockers, aldosterone antagonists, digoxin, anticoagulants, and inotropic agents [2]. For those who are refractory to medical treatment and develop end-stage heart failure, left ventricular assist system and heart transplantation are options but have limitations due to organ donation. With heart transplantation being the only definitive treatment, new therapies are continually being researched [3].

Stem cells have been an emerging trend in the research of heart failure treatment as they have the ability for self-renewal and have the potential to differentiate into different tissue types [4]. Stem cell therapy has the potential to replace and rebuild damaged myocardium and improve its function through neovascularization and the prevention of myocardial cell death [5]. 

Hope that one day stem cells will be a safe and efficient way to treat heart failure and improve the quality of life of patients, reduce mortality and morbidity, and decrease the global burden of this disease. For the practical application of stem cells to be clinically useful, a myriad of research must be done as there are many factors that need to be considered. Factors that need to be investigated and have been undergoing research are the type of stem cells used, application of the stem cells, dose, concentration, timing, patient population, and adverse outcomes. Safety concerns with regard to stem cell therapy in the myocardium are most related to increased incidence of ventricular arrhythmias [5]. While there has been much anticipation for the application of stem cells, research has so far been mixed. Skeletal myoblasts and bone marrow-derived stem cells are two common types of stem cells in heart failure therapy that are studied [4,5]. This literature review will focus on skeletal myoblasts and bone marrow-derived stem cells in improving LVEF in patients with heart failure.

## Review

Search strategy

Data for this review article were searched on PubMed using both regular and Medical Subject Heading (MeSH) keywords. The following keywords were used: stem cells, therapy, and heart failure. Search results were then narrowed down using inclusion/exclusion criteria. These criteria were based on language, species, year of publication, and subject age. Table 1 illustrates the search terms and the inclusion/exclusion criteria used along with the number of articles found. The inclusion/exclusion criteria were applied in the order that they appear in Table 1.

**Table 1:** Regular and MeSH Keywords and Inclusion/Exclusion Criteria Used in Literature Search MeSH: Medical Subject Heading

Criteria Used	Number of Articles Found
Regular Keywords – Stem Cells, Therapy, Heart Failure	2423
Inclusion/Exclusion Criteria	
English Language	2261
Human Species	1648
Published Within 5 Years	501
Aged 19+	97
MeSH Keywords – Stem Cells, Therapy, Heart Failure	371
Inclusion/Exclusion Criteria	
English Language	335
Human Species	275
Published Within 10 Years	143

Search result

A total of 2794 research articles was found using both the regular and MeSH keywords. After the results were filtered using the inclusion/exclusion criteria, a total of 240 articles remained. All 240 articles were reviewed and 208 were removed for the following reasons: duplication of articles (12), focused on a disease other than that of interest, and full text not freely available.

**Figure 1:**
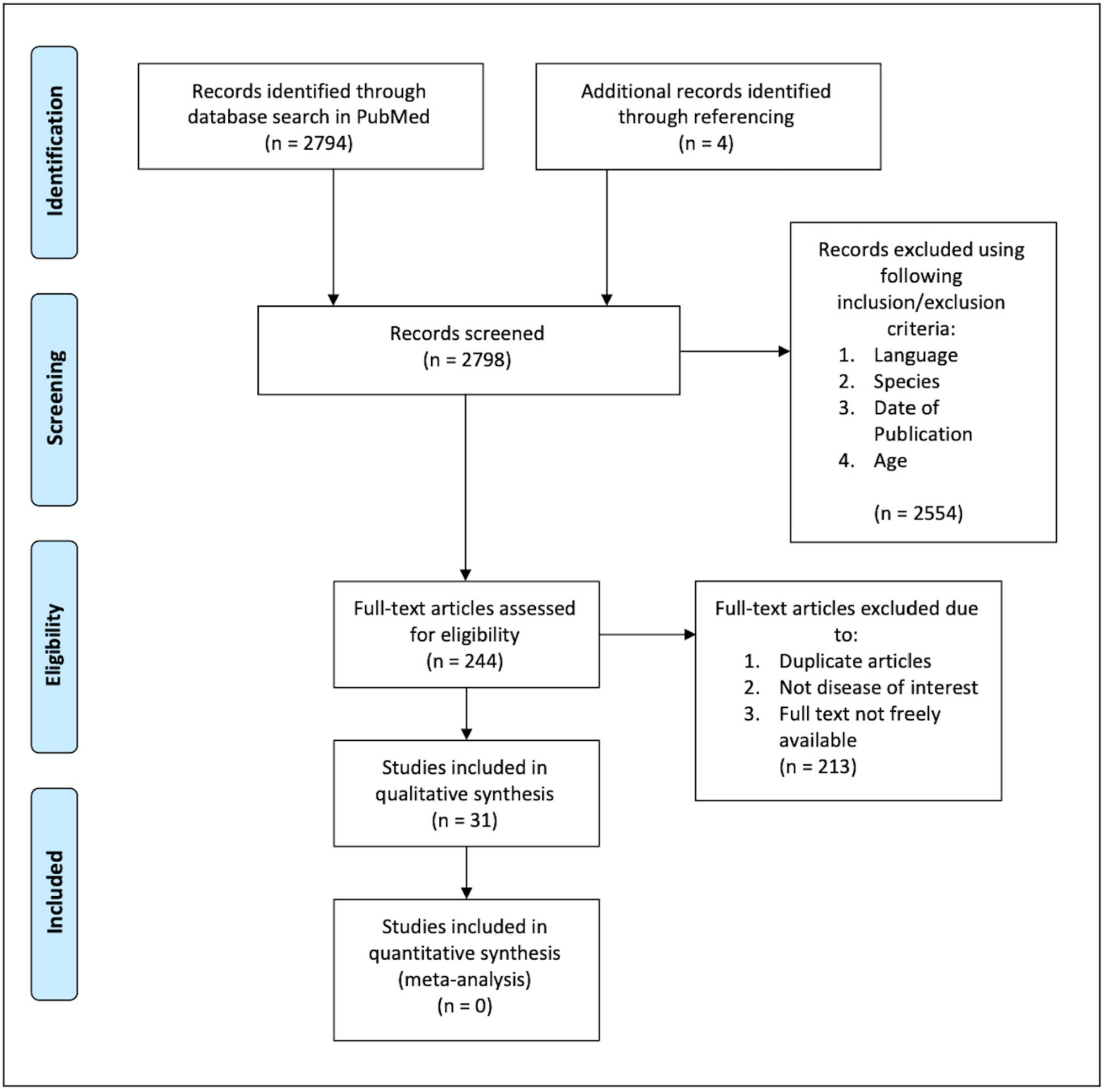
PRISMA Flow Diagram of Topic Search Results and Article Selection Process PRISMA: preferred reporting items for systematic reviews and meta-analyses

An additional four articles were manually selected through reviewing references of the selected articles. In total, 31 articles were reviewed and classified as follows: 14 were clinical trials, four were randomized control trials, nine were review articles, one was a systematic review, one was a case report, one was a comparative study and one was described as both a meta-analysis and systematic review. The minimum number of subjects in a study was one and the maximum was 2939 and the total number of subjects in the 31 reviewed articles was 5154. The process of data collection is shown below in the preferred reporting items for systematic reviews and meta-analyses (PRISMA) flow diagram in Figure 1 [6].

Discussion

After analysis of all data, it was found that neither type of stem cell has an overall positive effect in increasing LVEF. A total of six randomized control trials and five clinical trials involving either skeletal myoblast or bone marrow-derived stem cells for implantation in the myocardium of patients with heart failure were analyzed and tabulated. Other clinical trials that were analyzed examined repeated stem cell injection and stem cell transplantation in subjects with diabetes or insulin resistance. Two systematic reviews were also investigated to study the efficacy of stem cell therapy. It should be noted that while the studies used many endpoints to evaluate the effectiveness of their trial, this literature review focused on LVEF. 

Two groups of stem cells being studied with regard to heart failure are multipotent stem cells (adult stem cells) and pluripotent stem cells (embryonic stem cells or induced pluripotent stem cells). Multipotent stem cells can be isolated from various tissues such as skeletal muscle, adipose tissue, and peripheral blood or the bone marrow. An advantage of adult stem cells is that they can be used in autologous transplantation making them more accessible and without risk of immunological rejection [7]. Along with ethical and regulatory issues arising when dealing with embryonic cells, there is also the risk of malignancies occurring after implantation and for this reason, other types of stem cells remain a more attractive option [4].

The tables below show clinical trials that have been performed in ischemic and non-ischemic heart failure using either skeletal myoblasts or bone marrow-derived cells. The patient population for these trials was chosen using criteria such as LVEF and NYHA class.

Skeletal myoblasts

As mentioned before, skeletal myoblasts are an accessible source of autologous cells. They have also been shown to be resistant to ischemia, inflammation, and oxidative stress and able to form new myotubules in scarred myocardium in animal models [8]. As a result, skeletal myoblasts have been mostly studied in ischemic heart failure. Table 2 illustrates clinical trials that have studied the effects of skeletal myoblasts in heart failure.

**Table 2:** Summary of Clinical Studies Using Skeletal Myoblasts in Heart Failure HF: heart failure; LVEF: left ventricular heart failure; NYHA: New York Heart Association

Author/Year	Type of Study	Cells Used	Patient Population	Sample Size	Change in LVEF
Duckers et al., 2011 [8]	Clinical trial	Autologous skeletal myoblasts from quadriceps or gastrocnemius	Ischemic Cardiomyopathy with clinically manifest HF, LVEF = 20-45%, NYHA class ll-lll	47	LVEF at 6 months not statistically significant compared to control
Brickwedel et al., 2014 [9]	Clinical trial	Autologous skeletal myoblasts from upper thigh	Chronic ischemic heart disease with coronary artery bypass operation indicated, LVEF = 15-35%, NYHA class l-lV	7	No significant difference in LVEF across groups (high dose, low dose, placebo) at 12 months
Sawa et al., 2015 [3]	Clinical trial (no control group)	Autologous skeletal myoblast sheets (TCD-51073)	Ischemic heart disease with impaired left ventricular systolic function, LVEF = 35%, NYHA class lll-IV	7	LVEF maintained in 5 subjects and improved over time at 26 weeks
Gwizdala et al., 2017 [10]	Clinical trial (no control group)	Autologous Cx-43 modified skeletal muscle derived stem cells taken from quadriceps	Left ventricular dysfunction secondary to ischemic heart disease or dilated cardiomyopathy, LVEF =40%, NYHA class lll	13	No significant difference at 6 months

The data collected in Table 2 show that there has not been a success in increasing LVEF from autologous skeletal myoblast transplantation into the myocardium in ischemic heart failure. Sawa et al. did show an increase in LVEF in five of the seven subjects at 26 weeks but nothing else is known beyond this time frame and it was not measured against a control group [3]. Similarly, a review article done by Rikhtegar et al. found that in the long-term follow-up of the first phase l cohort study in severe heart failure, LVEF was shown to steadily improve over time after injection of skeletal myoblasts during a coronary artery bypass graft [11].

Bone marrow-derived cells

Like skeletal myoblasts, the adult bone marrow can be a great source of stem cells and these cells can be readily available and accessible for autologous transplantation without immune rejection. Bone marrow contains hematopoietic stem cells, endothelial progenitor cells, mesenchymal stem cells, and cardiac stem cells. Hematopoietic stem cells and endothelial progenitor cells can be isolated from the bone marrow cells through the identification of surface antigens such as CD34 [7]. Bone marrow-derived cells are promising in stem cell therapy as they have been shown to possess antifibrotic, proangiogenic, and immunomodulatory properties that can potentially stimulate the repair of damaged tissues [12]. Table 3 shows the main findings of the studies examined with regard to bone marrow-derived stem cells.

**Table 3:** Summary of Clinical Studies Using Bone Marrow-Derived Cells in Heart Failure LVEF: left ventricular ejection fraction; NYHA: New York Heart Association

Author/Year	Type of Study	Cells Used	Patient Population	Sample Size	Change in LVEF
Lezaic et al., 2015 [13]	Clinical trial (no control group)	Autologous CD34+ cells from peripheral blood by aphaeresis	Non-ischemic dilated cardiomyopathy, LVEF < 40%, NYHA class lll	21	10/21 showed significant changes in LVEF at 5 years
Hamshere et al., 2015 [14]	Randomized placebo-controlled trial	Autologous bone marrow-derived cells with granulocyte colony-stimulating factor (G-CSF)	Non-ischemic dilated cardiomyopathy, LVEF < 45%, NYHA class ll-lV	60	Statistically significant increase at 3 months and maintained at 1 year in the G-CSF/bone marrow cell treatment group
Butler et al., 2016 [15]	Randomized placebo-controlled trial	Ischemia tolerant allogeneic mesenchymal cells extracted from the bone marrow of young healthy volunteers	Non-ischemic cardiomyopathy, LVEF =40%, NYHA class ll-lll	23	No significant difference between groups at 90 days
Bervar et al., 2017 [16]	Clinical trial	Autologous CD34+ cells from peripheral blood by aphaeresis	Non-ischemic cardiomyopathy, LVEF < 40%, NYHA class lll	38	No significant differences between groups at 1, 3, 6, and 12 months (groups based on diastolic dysfunction with estimated filling pressures >/<15)
Teerlink et al., 2017 [17]	Randomized control trial (post hoc analysis)	Autologous mesenchymal bone marrow stem cells aspirated from the iliac crest	Chronic heart failure secondary to ischemic heart disease, LVEF < 35%, NYHA class ll-lV	315	No significant differences found in LVEF between groups at 1 year
Mostafavian et al., 2018 [18]	Clinical trial	Autologous mononuclear cells of bone marrow aspirated	Chronic heart failure, LVEF = 40%, NYHA class ll-lV	60	No significant difference between groups (treatment vs. control) at 3 months

The bone marrow-derived stem cell studies examined show slightly better outcomes compared to skeletal myoblast trials but were still unremarkable as a whole. Two of the studies were able to show improvement of LVEF > 5% at follow up. Lezaic et al. concluded that the results for the clinical trial showed improvement in 48% of their subjects at five-year follow-up but one of the limitations of the study was that they were not compared to a control group [13]. Hamshere et al. also saw a significant increase in LVEF at three-month follow-up and this increase was maintained at one year [14]. Similarly, in a post hoc analysis of three-phase l/ll clinical trials, it was found that bone marrow-derived cells in patients with non-ischemic dilated cardiomyopathy showed significant improvements in LVEF at 12 months both within their study group and compared to those with ischemic cardiomyopathy [12]. 

In addition to the studies in Table 3, two clinical trials using CD34+ peripheral blood cells in non-ischemic dilated cardiomyopathy were reviewed. These studies focused on repetitive injections and in diabetic and insulin-resistant patients, respectively. In the first study, results showed significant improvement in LVEF at six months having one dose but did not show further improvement at one year after a second dose was injected compared to the single-dose group [19]. In the second study, the patients with insulin resistance and no insulin resistance had a significant increase in LVEF while the diabetes group showed no improvement at six-month follow-up. It was noted that a higher dose of cells needed to be injected in the insulin-resistant patients for similar outcomes to the non-insulin resistant group showing a reduced efficacy in insulin resistance. Another important point was that the diabetic patients had more advanced heart failure than their counterparts. Whether the diabetes, the more advanced heart failure, or the combination of both played a role in seeing no change in LVEF will have to be further studied [20].

A systematic review that examined adult stem cell therapy studies between 2000 and 2016 found similar mixed results. One of these studies is the transplantation of progenitor cells and recovery of LV function in patients with chronic ischemic heart disease (TOPCARE-CHD) study, that showed significant improvement in LVEF at three months in ischemic cardiomyopathy using bone marrow-derived cells compared to circulating progenitor cells and placebo, but these results could not be replicated in a second study. Another one is the first mononuclear cells injected in the United States conducted by the cardiovascular cell therapy research network (FOCUS-CCTRN) study that also failed to show a significant increase in LVEF at six months after bone marrow-derived cell delivery [21]. This systematic review also examined a meta-analysis that included 23 randomized control trials using autologous bone marrow-derived cell delivery in ischemic cardiomyopathy also found no benefits at 12 months [21]. However, a study using autologous CD34+ cell transplantation in non-ischemic cardiomyopathy found a significant improvement in LVEF at both one and five years [21]. In a separate systematic review and meta-analysis, autologous bone marrow-derived stem cells did not prove effective in increasing LVEF in ischemic heart failure when compared to a control group [22].

Although there have been mixed results with studies showing LVEF improvement, it was found that there does not seem to be a significant increase in ventricular arrhythmias and these stem cells could be safely transplanted with regard to both skeletal myoblasts and bone marrow-derived cells [21,22]. While some of these results were discouraging it must be noted that the clinical trials were limited in size. The number of study data was also limited due to the inclusion/exclusion criteria used in the study method. Another reason for the discrepancy in results is that all trials are not created equal. There is a multitude of factors that are involved in these studies such as the cell source, cell type, cell application (dose, concentration, timing), and characteristics of the patient population. Study endpoints and how results are reported also differ between trials. Larger and more standardized future trials should be done as these could result in more definitive answers.

## Conclusions

This literature review of stem cells looked at their efficacy in increasing LVEF in heart failure patients. Skeletal myoblasts and bone marrow-derived cells are two types of stem cells that have been investigated in the treatment of heart failure. At best, mixed results were observed in the majority of studies showing that neither type of stem cell significantly improves LVEF in ischemic or non-ischemic heart failure. In order to further investigate the efficacy of stem cells in heart failure, more clinical trials with larger cohorts and endpoints should be conducted.
